# Scrotal Lipomatosis Mimicking Varicocele: A Case Report and Review of the Literature

**DOI:** 10.1155/2015/695314

**Published:** 2015-11-17

**Authors:** Sadi Turkan, Mehmet Kalkan, Coşkun Şahin

**Affiliations:** ^1^Private Anadolu Hospital, Department of Urology, Kastamonu, Turkey; ^2^Tıp Fakültesi Hastanesi, Fatih Üniversitesi, Sahil Yolu Sokak No. 16, Dragos, Maltepe, 34844 İstanbul, Turkey

## Abstract

Scrotal lipomatosis is a rarely seen disease with an etiology that is not fully understood. Some information suggests that this disease may be associated with infertility. It is characterized by pain-free scrotal swelling. In this study, we reported a scrotal lipomatosis case presenting due to infertility and pain-free scrotal swelling. It was operated on with the initial diagnosis of varicocele, but once fatty tissue was observed in the scrotum, the case was diagnosed as scrotal lipomatosis. Here, we present this rare case with a literature review.

## 1. Introduction

Scrotal lipomatosis, one of the diseases of the scrotal contents, is a rarely seen condition with an unknown etiology. It may be seen in association with Multiple Symmetrical Lipomatosis, which is also known as Madelung's disease. The scrotum is an uncommon localization for lipomatosis [1]. This rare condition may be confused with the more common diseases of varicocele and scrotal hernia [2, 3]. In addition, it is reported to be associated with infertility [4]. In this study, we describe a case presenting due to infertility and left scrotal swelling, which was operated on with the diagnosis of varicocele, but which was then, upon fatty tissue examination, given the definitive diagnosis of scrotal lipomatosis.

## 2. Case

**Figure 1:**
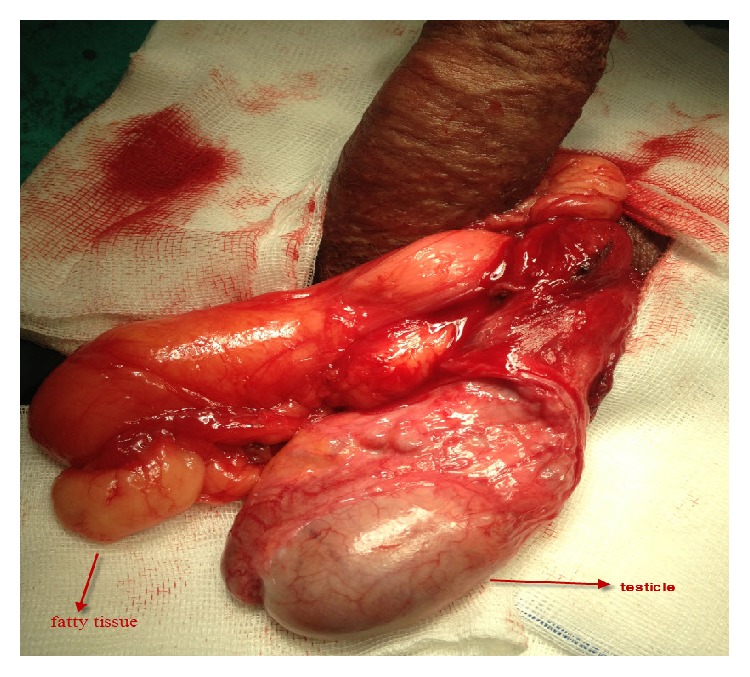
Observed fatty tissue beginning from the outlet of the inguinal channel and outside testicle into the scrotum in operative image.

**Figure 2:**
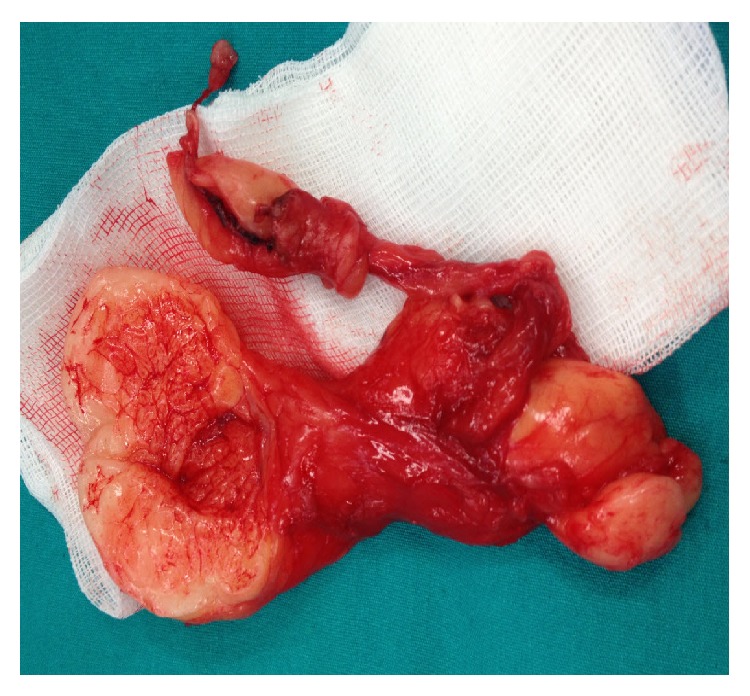
Fatty tissue that was completely excised.

A 34-year-old male patient who had had unprotected marital relations for two years presented to our clinic due to swelling in the left scrotum in addition to infertility. On physical examination, both the testicles were found to be localized in the scrotum with normal consistency and size. Soft tissues with elastic consistency were observed in the left part of the scrotum by means of transillumination, and these could not be reduced without pain. The presentation was thought to be compatible with Grade 3 varicocele. On nondestructive sperm analysis, the number of sperms was found to be 11 million/cc, the ratio of motile sperm within the first 30 minutes was found to be 15%, and the proportion with normal morphological structure according to the Kruger strict criteria was found to be 4%. The patient's body mass index (BMI) was 32 kg/m^2^. Upon exploration with a low inguinal incision, the soft tissue was understood not to be a varicocele but was observed to be a fatty tissue beginning from the outlet of the inguinal channel and outside testicle expanding into the scrotum (Figure 1). When the testicle and paratesticular tissues were removed, the testicle was found to be normal. The fatty tissue was readily separated from the testicle and the attachments and was completely excised (Figure 2). On pathological examination, it was understood to be a mature fatty tissue without observable cellular atypia or areas of necrosis. No pathology was seen in bilateral scrotal examination in postoperative second week. Control sperm analysis in postoperative 6 months was seen similar to preoperative values (the number of sperm: 11 million/cc, the ratio of motile sperm within the first 30 minutes: 15%, and normal morphological structure according to the Kruger strict criteria: 4%).

## 3. Discussion

Varicocele, hydrocele, testicular tumor, scrotal hernia, epididymitis, and orchitis are the diseases that are first thought of in the differential diagnosis of swelling within the scrotum. Varicocele is the disease most commonly presenting due to a palpable pain-free scrotal swelling, vasodilation complaints, or infertility [5]. Scrotal lipomatosis, defined as a fatty mass in the scrotum, is a condition that is rarely encountered in clinical practice. Therefore, this is commonly overlooked in differential diagnosis. Fatty tissues along the inguinal canal during herniorrhaphy have been loosely termed lipoma in the literature. However, it is very rare to observe a true lipoma. The lipoma or fat protrusion can present as an accompaniment to inguinal hernia. It is resected routinely during hernia repair and is rarely significant to the hernia sac as a groin bulge. While lipomatosis is able to infiltrate the surrounding tissue, lipomas are encapsulated [6]. Heller et al. demonstrated that inguinal canal lipoma is a common feature in the adult male population. They found no significant correlation between mass length and BMI [7]. Scrotal lipomas originating from mesenchyme may occur from spermatic cord, epididymis, tunica vaginalis, and subcutaneous fat cells of scrotum [8, 9].

Lipomas originating from subcutaneous fat cells of scrotum wall are termed scrotum primary lipoma. Origin of scrotal lipomas often can not be identified fully. Accordingly, the place of origin is divided into 3 groups: (1) originating from fat on the spermatic cord and extending towards the scrotum, (2) developing in the spermatic cord, and (3) originating from scrotal wall (primary scrotal lipoma) [8, 10].

When a literature search was performed using the key words “scrotal lipomatosis,” it yielded 14 articles, only 2 of which were directly related to scrotal lipomatosis. These patients present due to scrotal swelling or infertility. A spermatic cord lipoma may obscure an indirect inguinal hernia [11]. In the same way, in the literature, it was shown that scrotal lipomatosis in obese men may signify a distinct pathological manifestation of obesity involving the scrotum hindering varicocele detection [2]. Our patient also presented because of infertility. No clinical features were found in our patient's medical and familial history, other than his BMI of 32 kg/m^2^, indicating obesity. The soft, pain-free, elastic consistency of the tissue which was the content of the left scrotum was assessed as a Grade 3 varicocele on initial examination.

The most frequently reported cases of lipomatosis in the literature are those associated with multiple systemic lipomatosis. In this disease, fat aggregates are usually seen in the upper half of the body, neck, shoulder, and arms. Although more than 200 symmetrical lipomatosis cases have been reported, scrotal involvement has been observed in only a very small number of these patients [1, 3]. The etiology of this disease, which is usually seen from the 3rd to the 5th decade, is not fully known. The primary treatment of scrotal lipomatosis is lipectomy [12, 13].

Although extremely rare, it has been reported that scrotal lipomatosis might be associated with gynecomastia [14]. No gynecomastia was observed in our patient.

Two types of lipoidosis, classified as extratunical and intratunical, have been described as scrotal. It was reported that a small posterior extratunical pad of fat was constantly encountered in normal pattern. It was found also that intratunical fat occurred as small granules between the cord veins. They say that there is role of scrotal lipomatosis in subfertility and the relation of scrotal lipomatosis to obesity [2]. In other study, researchers claimed that scrotal lipomatosis causes primary idiopathic infertility [4].

Although normal lipoidosis is thought to be related to testicular temperature dysregulation, the relationship of the excessive lipoidosis in scrotal lipomatosis and infertility is not clearly known [12]. Because of the small number of known cases, it is not possible to conclude that infertility in our patient was caused by scrotal lipomatosis. A fuller understanding of the disease must await clinical reports of the treatment and follow-up results for a greater number of patients.

In conclusion, scrotal lipomatosis should be considered in the differential diagnosis of infertile men who present with pain-free scrotal swelling with a clearly palpable testicle and who are initially diagnosed with varicocele.
